# Supplementary figures and images for: Rifampicin exposure reveals within-host Mycobacterium tuberculosis diversity in patients with delayed culture conversion
Source: PLoS Pathog. 2021 Jun 24;17(6):e1009643. doi: 10.1371/journal.ppat.1009643 (PMC8224949; doi:10.1371/journal.ppat.1009643)

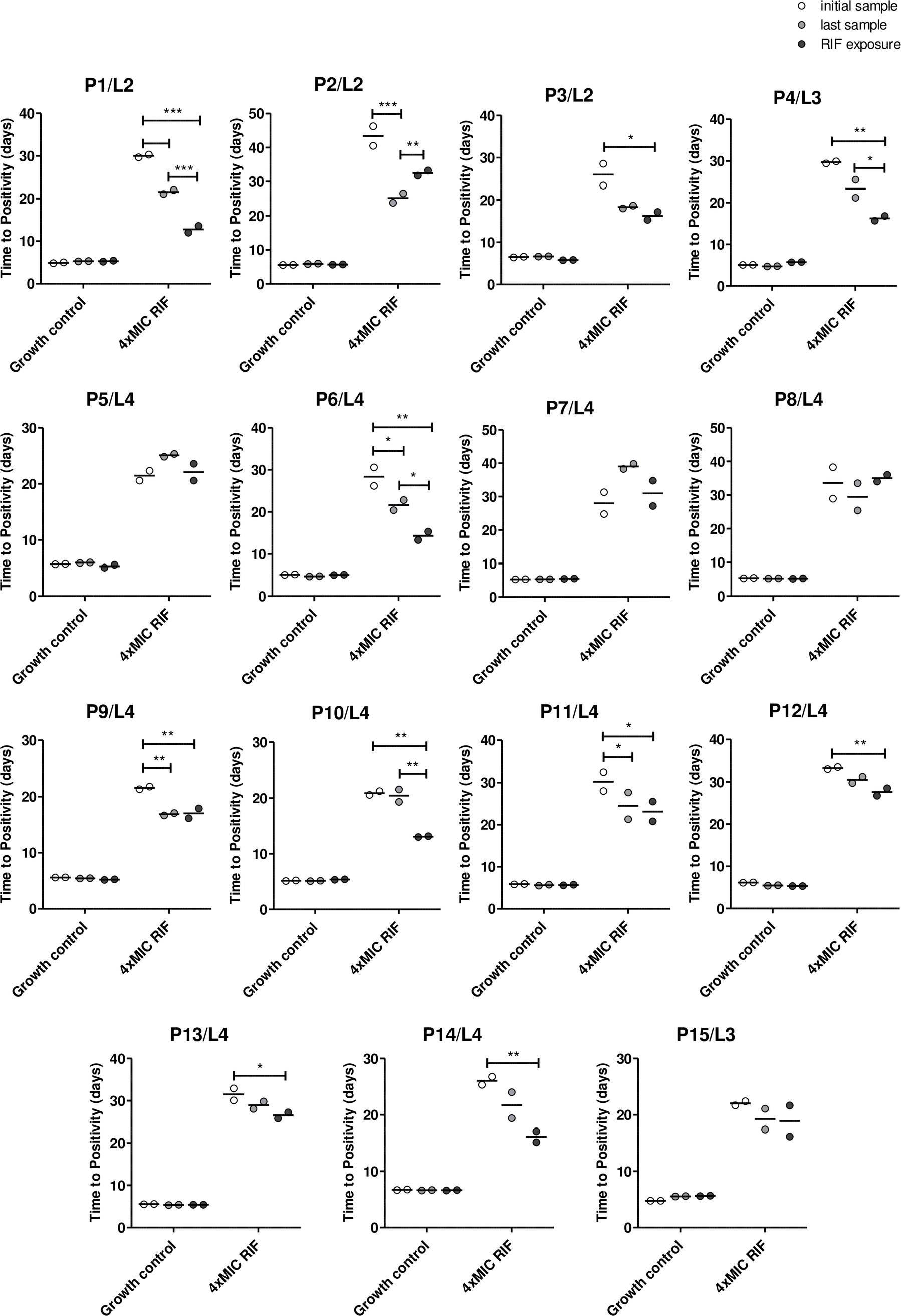

Supplement: S1 Fig — Mycobacterial growth of the initial isolate (white), last isolate (grey), and last isolate after 1xMIC RIF exposure (black) from patients with delayed culture conversion, in absence (Growth control, inoculated with 2.104 CFU/mL) or presence of RIF (black; 4x minimum inhibitory concentration (MIC) RIF, inoculated with 2.106 CFU/mL) is expressed in time to positivity (TTP) in the BACTEC system. Symbols represent values of two independent experiments. Means were compared using Repeated Measures ANOVA followed by Bonferroni correction. (TIF) [file ppat.1009643.s001.tif]

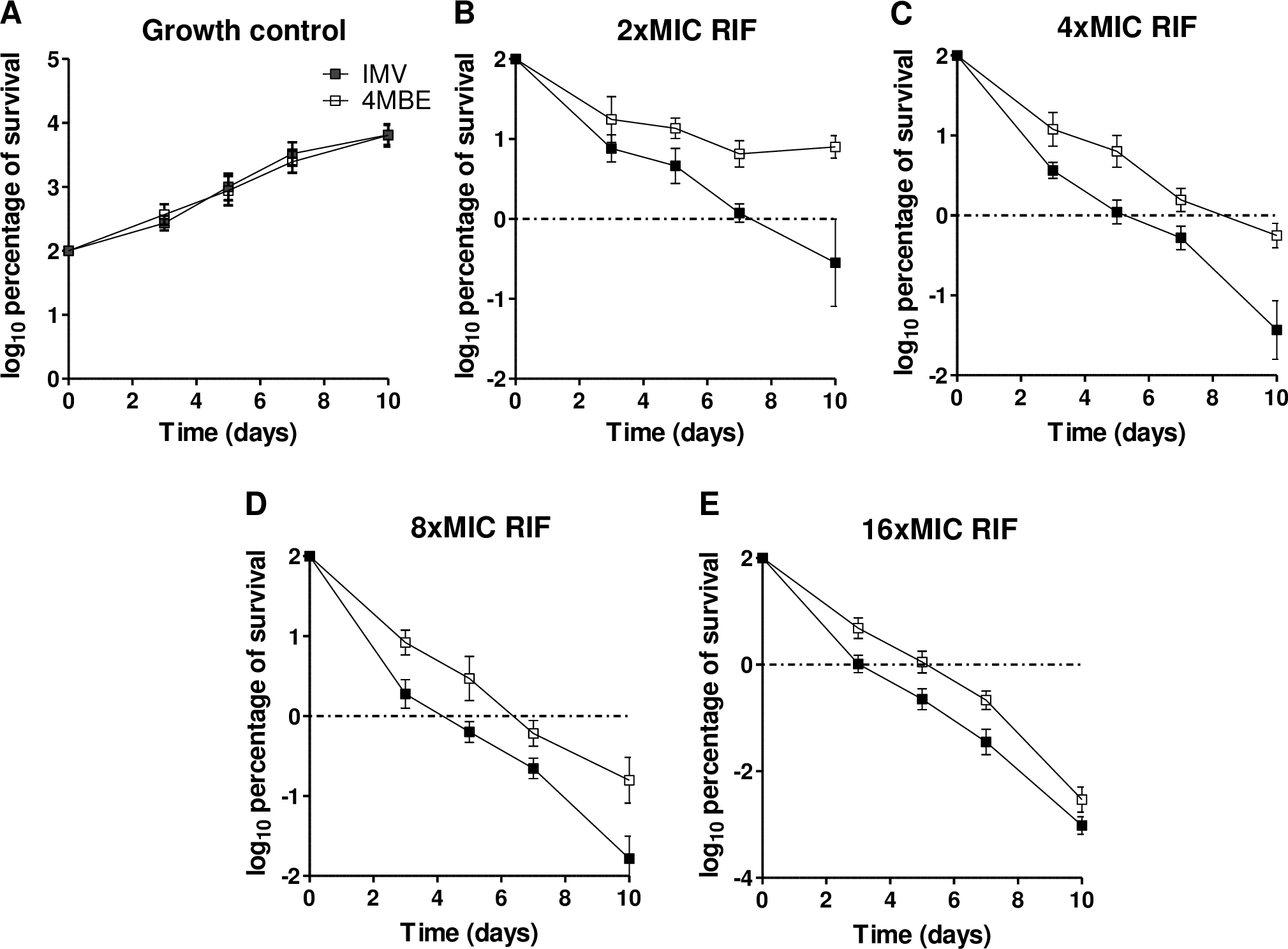

Supplement: S2 Fig — Mycobacterial growth of the IMV (black) and 4MBE variant (white) expressed as the percentage of survival obtained thanks to CFU count, without rifampicin (RIF) exposure (A, growth control), at 2xMIC (B), 4xMIC (C), 8xMIC (D) and 16xMIC (E) of RIF. The symbols represent the mean of three independent experiments; the bars represent standard deviation. (TIF) [file ppat.1009643.s002.tif]

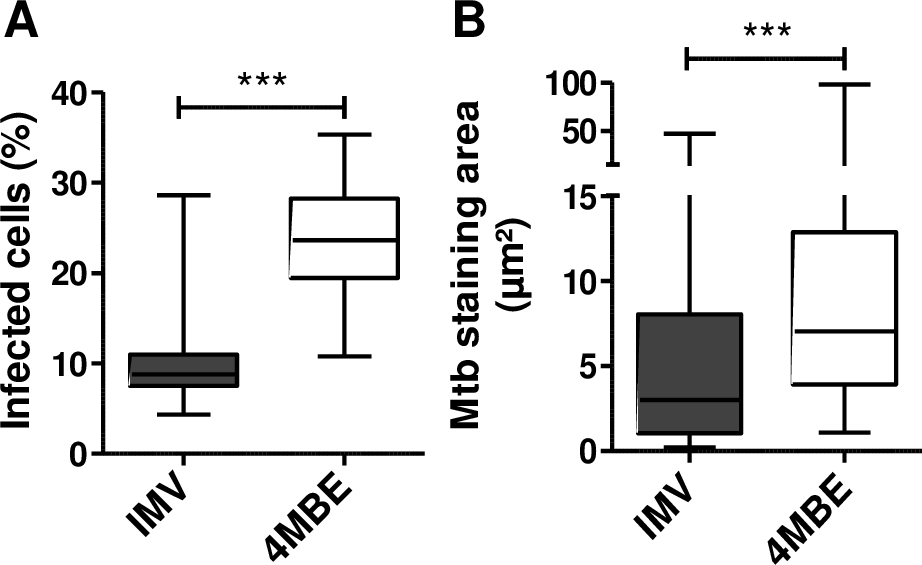

Supplement: S3 Fig — At 24 hpi, cells were stained with anti-Mtb coupled FITC. (A) The proportion of infected cells were determined across at least 10 confocal images, containing between 30 and 80 cells per image, per replicate. (B) Mtb staining area was determined across at least 40 cells per replicate. Values for each condition are the median values [interquartile range, IQR] of at least three independent experiments. Statistical significance was determined using Mann Whitney test. *p<0.05, ** p <0.01, *** p <0.001. (TIF) [file ppat.1009643.s003.tif]

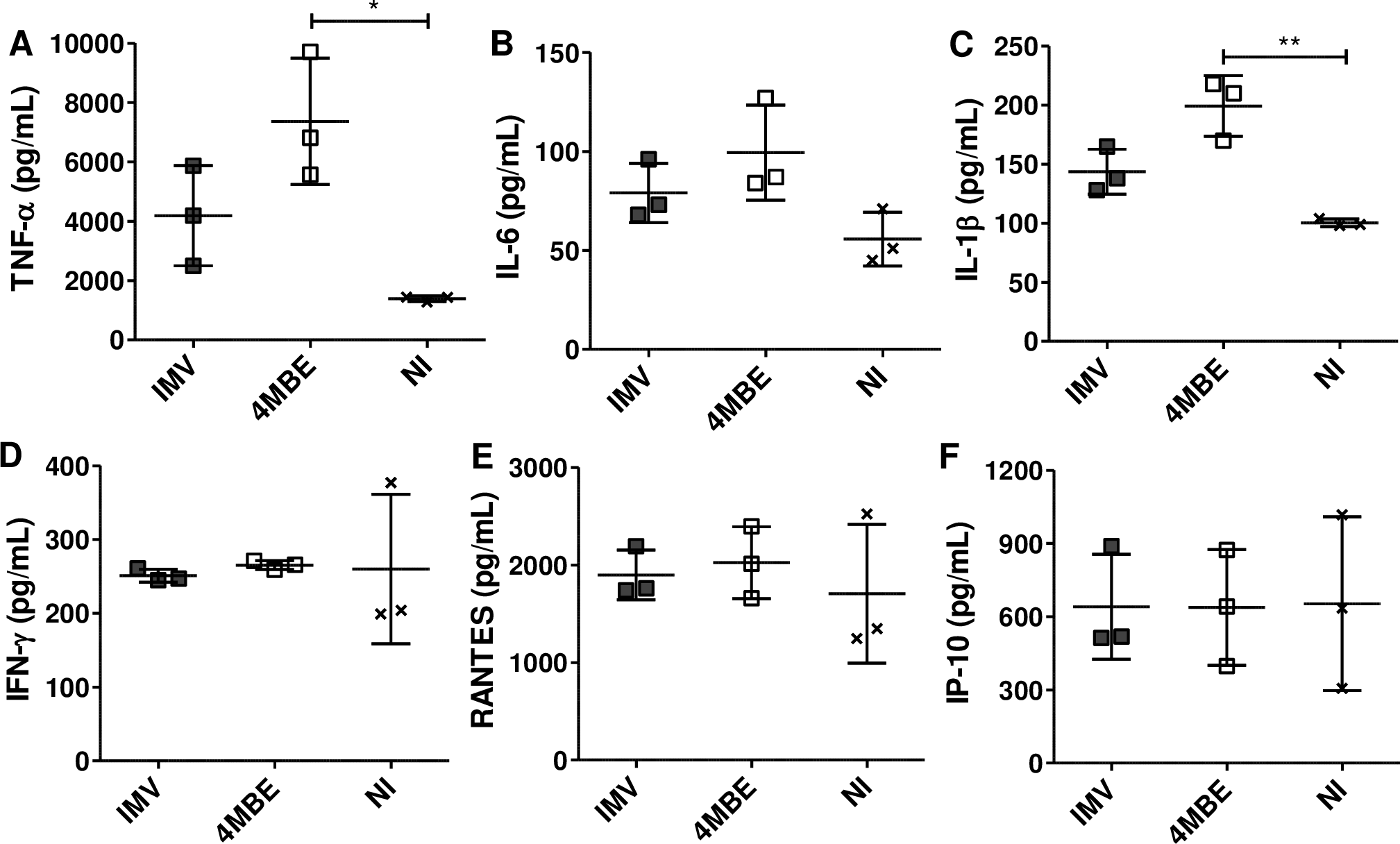

Supplement: S4 Fig — (A) TNF-α, (B) IL-6, (C) IL-1β, (D) IFN-γ, (E) CCL5 or RANTES and (F) Interferon gamma-induced protein 10 (IP-10) release in cell culture supernatant was evaluated at 6 hpi. Values for each condition are the mean ± standard deviation (SD) of three independent experiments. Means were compared using Repeated Measures ANOVA followed by Bonferroni correction. *p<0.05, ** p <0.01, *** p <0.001. (TIF) [file ppat.1009643.s004.tif]

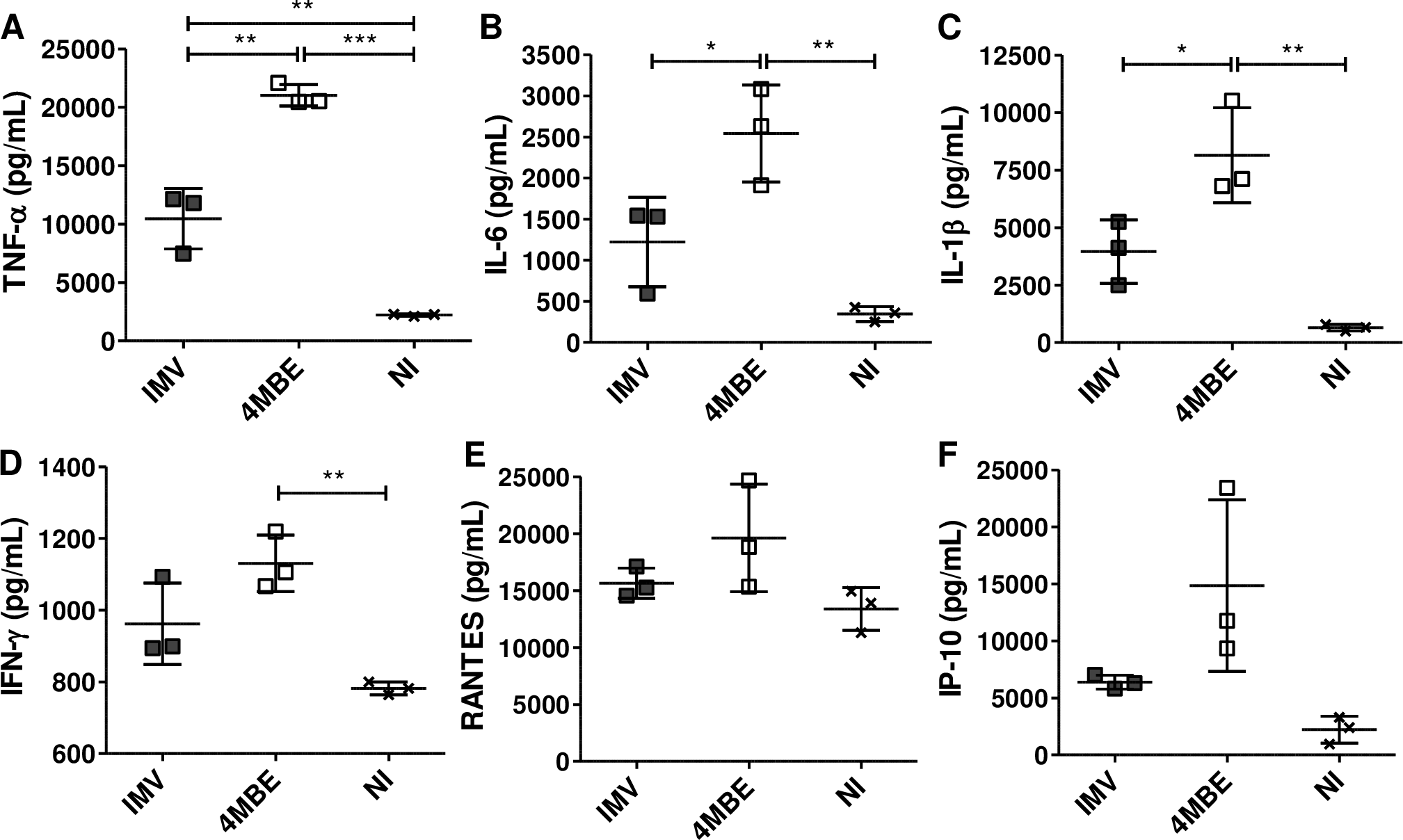

Supplement: S5 Fig — (A) TNF-α, (B) IL-6, (C) IL-1β, (D) IFN-γ, (E) CCL5 or RANTES and (F) Interferon gamma-induced protein 10 (IP-10) release in cell culture supernatant was evaluated at 96 hpi. Values for each condition are the mean ± standard deviation (SD) of three independent experiments. Means were compared using Repeated Measures ANOVA followed by Bonferroni correction. *p<0.05, ** p <0.01, *** p <0.001. (TIF) [file ppat.1009643.s005.tif]
