# Supplementary material for: Rifampicin exposure reveals within-host Mycobacterium tuberculosis diversity in patients with delayed culture conversion
Source: PLoS Pathog. 2021 Jun 24;17(6):e1009643. doi: 10.1371/journal.ppat.1009643 (PMC8224949; doi:10.1371/journal.ppat.1009643)
Supplement: S3 Table — (DOCX) [file ppat.1009643.s008.docx]

**S3 Table. Primer sets for targeted NGS**

| **Primer name** | **Sequence** | **Size (bp)** |
| --- | --- | --- |
| 3280555-variant-F | GTGTAGCAGTAGCGGGCATT | 1695 |
| 3280555-variant-R | AATCGGGATTCTCGGTGAC |  |
| 3281868-variant-F | TGGACGACAGCATGAATAGC | 1748 |
| 3281868-variant-R | TGCAACGGATGTAGTGCTTC |  |
| 4145334-variant-F | CGGTTAAATGCTCCTCGAAA | 1785 |
| 4145334-variant-R | ACGGTGAATGGCAAGACTTC |  |
| 2579217-variant-F | CGCGATAGTCAAACAGCAAC | 1671 |
| 2579217-variant-R | CTGAGGGACAACGATGACAG |  |
| 649763-variant-F | CGGTCTACCAAACCGATGTT | 1647 |
| 649763-variant-R | TACAAGTCGGCCACTTTCCT |  |
| 3849212-variant-F | GAGCAACCAACTCTGGCTTC | 1921 |
| 3849212-variant-R | CCTGATCAAAGCTCGTCGTC |  |
| P10-L4-kdpD-F | GGGGCTCGGTCAGAAAGTAGT | 803 |
| P10-L4-kdpD-R | CAACGTCTATGCAGCCGAAC |  |
| P10-L4-phoR-F | GTTGCAAATGCCATACAGCAC | 778 |
| P10-L4-phoR-R | TCGGTAACCAGGAATGAAACC |  |
| P10-L4-relK-F | TGTTTCCACTCATCGAACAGG | 937 |
| P10-L4-relK-R | TAGCGATCGGATTGAAGAACC |  |
| P12-L4-nuoD-F | GGAGGGGCTACAACTGATGAC | 792 |
| P12-L4-nuoD-R | AACCCAGTGGAACGCAGTATC |  |
| P12-L4-Rv0374c-F | GTACGTCGTTGGCTAGTGTCG | 787 |
| P12-L4-Rv0374c-R | GCAGGTGAACATGACGGTAAA |  |
| P12-L4-Rv1498c-F | ACGATGTCCTGGAAGCTTAGG | 848 |
| P12-L4-Rv1498c-R | GTATCTGGTCGACCTGTGTGG |  |
| P13-L4-Rv1226c-F | CGTCAGTTAGACCGTCGACAT | 865 |
| P13-L4-Rv1226c-R | GTTCTCCTGGTTGACCTACGG |  |
| P14-L4-2047599-F | GCTCTAGCGAGGCAAGAAAGA | 834 |
| P14-L4-2047599-R | CATGGGTCTTCTCCTGACAGA |  |
| P14-L4-888841-F | CTTCCGGTGCAGATGACAAC | 809 |
| P14-L4-888841-R | AAGCCACGCTCCTATCAAGTC |  |
| P14-L4-Rv2183c-F | GTTGATCCAGCCTTTCAGTCC | 961 |
| P14-L4-Rv2183c-R | GCCTATTGTGGACCTGGAATC |  |
| P14-L4-Rv2619c-F | CTGCGCAAGTACGGTTATGAA | 867 |
| P14-L4-Rv2619c-R | CAATACCGTTGAGGTCGGTTT |  |
| P15-L3-Rv1354c-F | GCGGTCCAAGTCGAGAAATAG | 818 |
| P15-L3-Rv1354c-R | GACTTTCGGGAACTGGTCAAG |  |
| P15-L3-suhB-F | CACCGAGCTGTATCACGACAG | 823 |
| P15-L3-suhB-R | ATTCTCGGGGAGGAAGGTG |  |
| P2-L2-Rv2568c-F | TCACACCCAGAAGAGTTGGAA | 799 |
| P2-L2-Rv2568c-R | GACACCAGAACGGAGTCATCA |  |
| P3-L2-cpsY-F | TGCTACACCCAGGACCTGAC | 881 |
| P3-L2-cpsY-R | GCTCAGCGAGCACTTCTTGTA |  |
| P3-L2-cysE-F | GCGAACGATATCTGAGCACAC | 745 |
| P3-L2-cysE-R | TTGCCTCAGATCGAGAAGTCC |  |
| P3-L2-pknB-F | CTGGAAAACGAAGTGCTCACC | 658 |
| P3-L2-pknB-R | GACGTCTCCACGCTGACATAC |  |
| P4-L3-hisA-F | GAGTGGATCCATTTGGTGGAC | 761 |
| P4-L3-hisA-R | GCTCGATCGCTAGATCGACTT |  |
| P4-L3-recB-F | GTCGGTAAGGCTCTCCTTGAG | 836 |
| P4-L3-recB-R | AGCAAGACCAGGAACCACATC |  |
| P4-L3-Rv0465c-F | CGTTTCGTAGACGTTCCACAG | 830 |
| P4-L3-Rv0465c-R | GCGATCTAGACATCGCCATC |  |
| P6-L4-ethA-F | CCGATCACGACGATGTTCTTA | 805 |
| P6-L4-ethA-R | TAGAACGTCGGCCTCGAGATA |  |
| P6-L4-hemA-F | CAGTATCGACGAATCCGATCA | 703 |
| P6-L4-hemA-R | AGTACAGCTGACCACCACGTC |  |
| P6-L4-Rv0272c-F | ACTTTGGCTTGCGGATCAT | 842 |
| P6-L4-Rv0272c-R | GTTCAGCGTGATGCGTACCT |  |
| P8-L4-miaA-F | GCAGAGTGCGTACCTCTTCAA | 737 |
| P8-L4-miaA-R | CCAAGGAGTTCTACCGAGGAG |  |
| P9-L4-moaA-F | CCCGTTATCGTTCCGTTATGT | 804 |
| P9-L4-moaA-R | GACCTGCTGAGGTTCTGCCTA |  |
| P9-L4-nrp-F | TCGATGTCGTCGATGCTTTAG | 814 |
| P9-L4-nrp-R | CGGATCTTGACCTGATCGTC |  |
| C7-L3-mutT1-F | GGCATTTCGGAAAACATCATC | 841 |
| C7-L3-mutT1-R | GCACAGGACTTTTCGATCCTG |  |
| C18-L4-atsB-F | GTGCGGTCTGGGTTGAACT | 816 |
| C18-L4-atsB-R | GACGGTCTGTATCGGCTGAC |  |
