# Supplementary material for: Rifampicin exposure reveals within-host Mycobacterium tuberculosis diversity in patients with delayed culture conversion
Source: PLoS Pathog. 2021 Jun 24;17(6):e1009643. doi: 10.1371/journal.ppat.1009643 (PMC8224949; doi:10.1371/journal.ppat.1009643)
Supplement: Attachment — Submitted filename: PPATHOGENS-S-20-03043_Response to reviewers_20210505.docx [file ppat.1009643.s009.docx]

| Director : François-Loïc Cosset  **Deputy Director : François Vandenesch**  **Charlotte Genestet**  **Centre International de Recherche en Infectiologie**  charlotte.genestet@gmail.com |  |
| --- | --- |

Lyon, May 5, 2021

To Plos Pathogens Editors

Dear Editors,

Please find enclosed the revised manuscript entitled “Rifampicin exposure reveals within-host Mycobacterium tuberculosis diversity in patients with delayed culture conversion”, that we are resubmitting for consideration in Plos Pathogens.

We thank the reviewers for their relevant comments and suggestions, and below is a point-by-point rebuttal (in bold).

We hope you would find our rebuttal arguments convincing and accept this revised manuscript for publication in Plos Pathogens, but we remain at your disposal if any further modifications are required.

Yours sincerely,

Charlotte Genestet

Corresponding author

Section Editor:

The reviewers raised important points that require additional work. The authors should particularly focus on performing the drug tolerance experiments to get a true readout of tolerance as well as including isolates from other patients in the same cohort as well as isolates from a control cohort. Thus, the drug tolerance experiments need to be done properly so as to get a more accurate readout of tolerance. When repeating this work, it would be advisable to include isolates from other patients in the delayed culture conversion cohort and control isolates from patients in a cohort without delayed culture conversion. Sequencing of the isolates from the patients without delayed culture conversion would provide the necessary sequencing data to ascertain how common the reported minor genetic variation really is.

**Authors: We agree that the experiments performed in this manuscript to explore antibiotic tolerance were not the conventional ones. Therefore, as requested, standard tolerance experiments were performed, and a panel has been added to Fig 4. These experiments allowed to confirm that the 4MBE variant is more tolerant to RIF than the IMV. The Results section has been revised accordingly (lines 231-239) as well as the Material and Methods section (lines 536-540).**

**As requested, three other patients with delayed culture conversion were added to the cohort. As we fixed a 2-year delay after treatment completion for “cure without relapse” assessment, we were able to retrospectively select isolates up to 2017, and as isolates were kept on slants and not frozen before 2014 we could not use isolates collected before this date. The Results section (lines 121-127 and 140-145), Figure 1, Table 1, Discussion section (lines 349-360) and the Material and Methods section (lines 414-428) have been modified to include the results obtained with these additional isolates.**

**Regarding the control cohort, we agree that control isolates from patients without delayed culture conversion are relevant to support the conclusions made. Accordingly, we have added 20 new patients with AFB+ pulmonary TB with proven culture conversion before 2 months of anti-TB treatment. Regarding initial isolates of the control cohort, 4/20 (20%) presented at least 1 unfixed mutation, among which 2 nsSNP,** **and 2 additional nsSNP from 2/20 (10%) isolates were revealed by RIF exposure. These results indicate** **a lower proportion of minor genetic variation in the control cohort compared to the delayed culture conversion cohort, supporting our conclusions. Table 2, summarizing patients and microbiological data, has been added to the revised manuscript. The Results section (lines 127-131 and 137-140), Discussion section (lines 350-352 and 362-364) and the Material and Methods section (lines 418-428) have been revised accordingly.**

Reviewer #1:

Part I - Summary

The study aims to provide insights in the evolution of M. tuberculosis in the host with a particular focus on delayed treatment response, resistance development, and antibiotic tolerant sub-populations. The used a combination of microbiological analysis with WGS to unravel mutant populations in the initial cultures that may be enriched during therapy.

1) I am a bit uncertain about the experimental procedures. On particular point is that the antibiotic tolerant phenotype has not genetic trait, so it is reversible. How can this then be captured?

**Authors: We did not observe any reversion of the tolerant phenotype during the different experiments conducted. In addition, the tolerant phenotype was discriminated, according to WGS short-read data, by the C 3280555 G SNP. This genetic trait was used to capture its evolution by ddPCR during controlled competition experiments (Fig 4 and 5 of the initially submitted manuscript). The polymorphism targeted in ddPCR experiments has been clarified in the Results section (lines 257-258)**

2) Also, is there any evidence that the pre-treatment with 1 x MIC of RIF is the right dosage for pre-selection?

**Authors: During protocol optimization two other doses were tested, 0.5 and 2 x MIC. However, the lower dose failed to unmask new variants in some experiments. Conversely, with the higher dose we observed in some experiments, the emergence of mutations in canonical drug resistance determinants, which was not the aim of this study, as these mutations in *rpo*B locus were responsible of drug resistance and not tolerance.**

3) While the paper is well written overall, however, several points need to be addressed in a revised version. This is esp. the case for the selection and macrophage experiments. How many times have they been done? It there any statistics done demonstrating that this is not chance?

**Authors: Experiments of RIF exposure on the isolates from the delayed culture conversion cohort were performed once, and footnotes have been added to Table 1 to specify this (lines 158-159). No statistical analysis was therefore conducted, but the experiments of targeted NGS performed in the revised manuscript allowed to demonstrate that all the mutations revealed by RIF exposure were found in the paired last isolates, supporting that this was not by chance.**

**Regarding experiments focusing on the P1/L2 isolate, two independent experiments were performed to demonstrate that the selection of this particular variant during stress experiments (RIF exposure and macrophage infection) is not by chance and the legend to Fig 2 has been clarified.**

4) The variant detected is quite interesting, if this is really providing selective advantage in case of RIF treatment, the variant should be seen in clinical isolates already. This should be checked as genome data are available from a high number of clinical isolates.

**Authors:** **We did screen public databases (****ReseqTB, phyTB, GMTVD) to evaluate the prevalence of this mutation in clinical isolates before the initial submission. Unfortunately,** **the quality of available archives is often insufficient to detect minor variants. Therefore, we screened our own clinical isolates by ddPCR for in-depth analysis. We were able to detect this variant, at a frequency (~1%) undetectable by WGS, in another Mtb clinical isolate. Among the 140 clinical isolates tested by ddPCR screening, this particular isolate was multi-drug resistant (MDR) belonging to the Euro-American lineage. Accordingly, a paragraph has been added to the Discussion section (lines 373-379). Although the C 3280555 G SNP may be linked to RIF tolerance, we provided in the discussion section in the initially submitted manuscript an explanation to support the fact that C 3280555 G SNP has not yet been detected as a fixed SNP in other strains (i.e. the pro-inflammatory profile of the variant could mainly favor infection initiation, and be less prone to develop in long-term infection, explaining its low frequency in clinical isolates).**

Part II – Major Issues: Key Experiments Required for Acceptance

5) Line 77 - 80 – please include references for the statements

**Authors: As requested, references have been added (line 86).**

Results

6) Line 122 – it is important to detail the experimental procedures – what was used for DNA extraction for the culture, how deep was sequenced? Also, how were the minor variants been identified? Any particular statistical method for that?

**Authors: Genomic DNA was purified from cleared lysate of whole bacterial culture, obtained after lysozyme and proteinase K digestion, using a QIAamp DNA mini Kit (Qiagen). The WGS reference genome coverage ranges 96.5% to 99.0%, with an average depth of coverage of 46x to 185x as stated in the Material and Methods section of the initially submitted manuscript. Following this comment, the Material and Methods section (line 491 and lines 500-506) has been further clarified and footnotes have been added to Table 1 (lines 158-160) to further detail the experimental procedures.**

**Minor variants were identified thanks to unfixed mutations observed by WGS (initially submitted manuscript) and targeted NGS (revised manuscript). This has been clarified in Results section (line 133).**

**No statistical method per se has been carried out, but to ascertain the robustness of the detection of minor variants, we performed experiments that found less than 10% variation of unfixed mutation frequencies between sequencing experiments (see Figure below). This figure can be added to the manuscript in the supporting information if the editor would like us to do so.**

**Figure: Evaluation of variations of unfixed mutation frequencies between sequencing experiments.**

**Ten isolates, containing 52 unfixed mutations at frequencies ranging from 10% to 90%, were extracted and sequenced twice to evaluate the variability in mutation frequencies between experiments. Continuous line is the linear regression curve. Discontinuous bands are the 90% prediction bands.**

7) Line 123 – how was the RIF pretreatment done? The overall procedure is not really clear to me, but hast a strong influence of the outcome. How many repetitions did you do? Any statistics done?

**Authors: The last isolates of each patient of the delayed culture conversion cohort were exposed *in vitro* to RIF at 1xMIC in MGIT for 4 weeks prior to WGS. Experiments of RIF exposure on the isolates from the delayed culture conversion cohort were performed once, and footnotes have been added to Table 1 to specify this (line 158). No statistical analysis has been done here, but the experiments of targeted NGS performed in the revised manuscript allowed to demonstrate that all the mutations revealed by RIF exposure were found in the paired last isolate, supporting our conclusions. Following this comment this sub-section has been further clarified (line 144) and footnotes have been added to Table 1 (lines 158-160) to further detail the experimental procedures.**

8) Line 137 – please explain a bit more precisely what you did – how was the single colony plating done? Was it from the initial culture or the subsequent one?

**Authors: Bacterial suspension of the last isolate of P1/L2 patient was homogenized by vortexing in the presence of glass beads and Tween 80, and were plated on 7H10 supplemented with OADC to obtain single colonies. The Material and Methods section (lines 432-435) has been clarified, as well as the first paragraph of the Results sub-section “A minor variant of Mtb clinical isolate is selected in response to both rifampicin exposure and macrophage infection” (lines 174-175).**

9) Line 139 - targeted NGS of what? How was this done?

**Authors: The targeted NGS was performed on the last isolate of P1/L2 patient, before or after stress experiments (RIF exposure or macrophage infection). After 4 weeks of *in vitro* RIF exposure at 1xMIC in MGIT, bacteria were plated and then sequenced. For macrophage infection, macrophages were infected by the last isolate of P1 patient at a multiplicity of infection (MOI) of 10:1 (bacteria:cells). At 7 days post-infection, macrophages were lysed to recover, plate, and then sequence intracellular bacteria. The Results sub-section “A minor variant of Mtb clinical isolate is selected in response to both rifampicin exposure and macrophage infection” (lines 177 and 180-181) and Figure 2 legend (lines 942-949) have been clarified.**

10) Line 144 – the macrophage experiments are not clear to me. How long are they performed? How many generations of mycobacterial growth would you expect? Please provide growth curves etc. Even if experiments are done for one week – the number of generations for potential selection of the variant is really low. So, please provide more data on it. Also, did you do the experiments in repetition to so that this does not happen by chance? Also, did you check the inoculum and/or the pre-culture for the frequencies?

**Authors: Macrophages were infected by the last isolate of P1 patient at a multiplicity of infection (MOI) of 10:1 (bacteria:cells). At 7 days post-infection, macrophages were lysed to recover, plate and then sequence intracellular bacteria. Following this comment, the Results section (lines 180-181) and Figure 2 legend (lines 942-949) have been clarified.**

**Regarding the growth of bacteria during the 7 days of macrophage infection at a MOI of 10:1, we observed about 40 times more bacteria after 7 days than on day 0 (see Figure below). But, more importantly, we showed in Figures 5 and 6 of the initially submitted manuscript that the significant increase in the frequency of this variant in such a short time was not only due to a better multiplication of the 4MBE variant compared to the IMV, but at least also thanks to a better intracellular survival.**

**Regarding the repetition of experiments, two independent experiments of macrophage infection were performed to ensure that the selection of this variant does not happen by chance. This point has been clarified in the legend to Figure 2 (lines 948-949). Moreover, macrophage infection with a 50:50 mixture of the IMV and 4MBE variant revealed a significant enrichment of the 4MBE variant (p=0.0022), up to 75.0 ±10.7% at 96 h post-infection, consistent with these targeted NGS experiments (Figure 5C in the initially submitted manuscript).**

**The frequency of the 4MBE variant in the last isolate of the P1 patient was checked by targeted NGS, as illustrated in Figure 2A (initially submitted manuscript) and in Table 1 (revised manuscript). Moreover, the 4MBE variant frequency was also evaluated in the last isolate of P1 patient by ddPCR, showing a frequency between 2 and 3% of this variant in the initial inoculum (data not shown).**

**Figure: Mtb multiplication during the 7 days of macrophage infection**

**Macrophages were infected by the last isolate of P1 patient at a MOI of 10:1. At 6 hours and 7 days post-infection, total bacteria and intracellular bacteria were plated for CFU counting. Bacterial multiplication was assessed thanks to CFU ratio between day 7 and day 0 (6 hours post-infection).**

11) Line 149 – what do you mean with “cloned variants”.

**Authors: “Cloned variants” means “single-colony variants” stemmed from the clinical isolate. This point has been clarified in the Results section (lines 187-188).**

12) Line 147 to 153 – I did not understand what was done here, please clarify

**Authors: Bacterial suspensions of the last isolate of P1/L2 patient was homogenized by vortexing in the presence of glass beads and Tween 80, and were plated on 7H10 supplemented with OADC to obtain single colonies. Clones were submitted to WGS to confirm the absence (IMV) or presence (4MBE) of the polymorphism of interest (C 3280555 G) and to confirm the absence of other mutations within the limits of the interpretation of short-read sequencing. The Material and Methods section (lines 433-435) has been clarified, as well as the second paragraph of the Results section “A minor variant of Mtb clinical isolate is selected in response to both rifampicin exposure and macrophage infection” (lines 187-188).**

13) Line 166 – what do you mean with significantly here – did you do statistics? If yes, include values.

**Authors: As shown in the Fig 3B of the initially submitted manuscript, the p-value is < 0.001. Following this comment, the Figure legends have been clarified.**

14) Line 181 – difficult to believe that if the MIC is not changed, the growth in 1x MIC is better for the mutant. Indeed, now growth should be observed if the strains are treated with 1 x MIC of RIF - or? So, either the MIC finding is wrong – or the time to culture positivity data make no sense.

**Authors: The inoculum in the control MGIT is 100 times lower than in RIF exposure MGIT, as stated in the Material and Methods sub-section “Antibiotics exposure using the BACTEC 960 system” of the initially submitted manuscript. Both IMV and 4MBE variants grew later than the control, confirming that they had the same MIC. Besides, MIC does not indicate the “no growth” concentration, but “the absence of visible bacterial growth”. Two bacterial strains may therefore share a MIC but have different killing rates when submitted to the same antibiotic. Following this comment, the Figure 4 legend (lines 967-968) has been clarified regarding bacterial inoculum for control and RIF exposure experiments.**

15) Also, general drug tolerance experiments are done differently. You need to expose a culture with high dose treatment and then do CFU over time to measure the treatment refractory proportion of the culture. This should be done for the mutant clone and the parental strain to see if there is really a higher drug tolerance level.

**Authors: We agree that the experiments performed in this manuscript to explore antibiotic tolerance were not the conventional ones. Accordingly, a panel has been added to Fig 4 presenting the results of standard tolerance experiments. These experiments allowed to confirm that the 4MBE variant is more tolerant to RIF than the IMV. The Results section (lines 231-239), as well as the Material and Methods section (lines 536-540) have been revised accordingly.**

16) Line 219 – do you mean uptake?

**Authors: Since we have not demonstrated whether this was an active mechanism of Mtb entry into macrophages, the term “invasion” has been revised to “uptake” throughout the manuscript.**

Discussion

17) General: In host diversity has been described before, so I do find the data presented here add little further knowledge. However, the 4MBE variant is really interesting. If the overall advantages of the variant are as drastic as described, it should already be found in nature. As all the experiments performed in vitro, it would be great if the authors can screen WGS data collections for the presence of the variant in clinical isolates.

**Authors: Strengths of this manuscript are the fact that it includes both clinical and genetic data, and then explores functional consequences of the variant, which is often not done by equivalent papers. Moreover, we did screen public databases (ReseqTB, phyTB, GMTVD) to evaluate the prevalence of this mutation in clinical isolates before the initial submission. Unfortunately, the quality of available archives is often insufficient to detect minor variants. Therefore, we screened our own clinical isolates by ddPCR for in-depth analysis. We were able to detect this variant, at a frequency (~1%) undetectable by WGS, in another Mtb clinical isolate. Among the 140 clinical isolates tested by ddPCR screening, this particular isolate was multi-drug resistant (MDR) belonging to the Euro-American lineage. Accordingly, a paragraph has been added to the discussion section (lines 373-379).**

18) The tolerance phenotype is not really clear to me, as the authors did not really perform drug tolerance experiments.

**Authors: As stated above, conventional tolerance experiments were performed as requested to confirm the tolerant phenotype and the revised manuscript has been modified accordingly.**

19) It would also be great to get an idea about the mechanism.

**Authors: We performed preliminary experiments to explore mechanisms often involved in drug tolerance, such as the efflux pumps (see below). These figures presenting the results of these experiments, involving efflux pumps inhibitors’ effect, can be added to the revised manuscript at editor request.**

**Figure: Efflux pumps are involved in RIF tolerance of the 4MBE variant.**

**(A). IMV and 4MBE variant were incubated with either reserpine (100 µM) or valinomycin (0.1 µM) in the absence or presence of RIF at 0.06 mg/L or 0.12 mg/L, or without efflux pump inhibitor as control. Results are expressed in delta of time to positivity (∆TTP) between cultures in presence and absence of efflux pump inhibitor.** **Values for each condition are the mean ± SD of at least three independent experiments. Means were compared using one-way ANOVA followed by Bonferroni correction (B). Quantitative RT-PCR were performed and normalized to 16S rRNA from the same sample. These results represent the differences in the mRNA levels detected in 4MBE variant relative to the mRNA level of IMV upon RIF exposure at 1/8xMIC. Values for each condition are the mean ± SD of at least three independent experiments. Means were compared using Student’s t-test.**

Reviewer #2:

Part I - Summary

The authors run a screening on several isolates from patients with delayed culture conversion, sequencing both the first and the last isolates coming from those patients. They also grow the last isolates from the patients in the presence of an inhibitory dose of rifampicin and sequence the resulting bacterial population. They find a series of variant mutations and decide to study one patient in particular. They manage to isolate single colony variants from this patient, one carrying one potentially interesting mutation (4MBE variant) and one identical to the initial majority variant in the patient (IMV). They demonstrate that the 4MBE variant has selective advantage over the IMV in the presence of rifampicin even if they have the same MIC and that the 4MBE variant has increased ability to infect macrophages and replicate or survive intramacrophagically, and the variant increases apoptosis and inflammatory response in macrophages.

1) The authors are aiming to establish a link between micro-diversity in clinical isolates and delayed culture conversion by means of low-frequency variants with increased tolerance for the rifampicin and increased persistence in macrophages. They study one low frequency variant from a patient with delayed culture conversion and convincingly demonstrate that the variant has selective advantage both in the presence of rifampicin and in the infection of macrophages (in vitro). However, I do not think that the main claims of the paper are supported by the evidence as presented. This is not because their conclusions are necessarily incorrect per se, but rather because their results are insufficient to support those conclusions. Although the authors claim to be studying within-host evolution, most of the paper is focused on a single patient with no clear rationale for doing so and the data from the rest of the cohort are very basic and not explored further. In my opinion, they should either re-frame the paper or find additional evidence.

**Authors: In order to better support the main claim of the paper, additional experiments were performed. Please find the in-depth answers to your suggestions in the point-by-point rebuttal below.**

Part II – Major Issues: Key Experiments Required for Acceptance

2) The authors do not sequence or present any data from patients without delayed culture conversion. If they are arguing that the micro-diversity found in those patients is relevant we would need a control cohort for comparison. We would need a rifampicin exposure experiment with those control isolates to show that samples from delayed culture conversion patients have more new variants than the controls.

**Authors: We agree that control isolates from patients without delayed culture conversion are pertinent to ascertain the conclusions made. Accordingly, a control cohort of 20 new patients with AFB+ pulmonary TB with proven culture conversion before 2 months of anti-TB treatment has been added. Regarding initial isolates of the control cohort, 4/20 (20%) presented at least 1 unfixed mutation, among which 2 nsSNP, and 2 additional nsSNP from 2/20 (10%) isolates were revealed by RIF exposure. These results indicated a lower proportion of minor genetic variation in the control cohort compared to the delayed culture conversion cohort, supporting our conclusions. Table 2, summarizing patients and microbiological data, has been added and the Results section (lines 128-131 and 137-140) was revised accordingly.**

3) The authors find several mutations that appear only after exposure to rifampicin in several patients, but they do not go back and try to find if these mutations were in the patients before the exposure using targeted sequencing. Furthermore, the authors do not show any evidence that these mutations (particularly the one in the variant they focus on) are not present in patients with early culture conversion or sequence isolates from any such patients for comparison. Also, if the mutation in 4MBE is responsible for the late culture conversion of the patient and has (limited) selective advantage in the presence of rifampicin and confers some advantage in macrophage infection, why is it such a minority variant in the isolates from the patient?

**Authors: We agree that target sequencing experiments are needed to support the conclusion made. As suggested, targeted NGS was performed to investigate identified mutations in other isolates of the same patient. Among the 9 unfixed nsSNP revealed by RIF exposure in 6/15 isolates, all were detected at low frequency thanks to targeted NGS in the last isolate, supporting our conclusions. Results of targeted NGS have been added to Table 1.**

**Also, we did screen public databases (ReseqTB, phyTB, GMTVD) to evaluate the prevalence of this mutation in clinical isolates before the initial submission. Unfortunately, the quality of available archives is often insufficient to detect minor variants. Therefore, we screened our own clinical isolates by ddPCR for in-depth analysis. We were able to detect this variant, at a frequency (~1%) undetectable by WGS, in another Mtb clinical isolate. Among the 140 clinical isolates tested by ddPCR screening, this particular isolate was multi-drug resistant (MDR) belonging to the Euro-American lineage. Accordingly, a paragraph has been added to the Discussion section (lines 373-379). Though the C 3280555 G SNP may be linked to RIF tolerance, we provided in the Discussion section in the initially submitted manuscript an explanation to support the fact that C 3280555 G SNP has not yet been detected as a fixed SNP in other strains (i.e. the pro-inflammatory profile of the variant could mainly favor infection initiation, and be less prone to develop in long-term infection, explaining its low frequency in clinical isolates).**

4) It is unclear to me why the authors decide to focus on a particular patient and not any of the others. They should explain better why they chose to focus on this patient specifically. If there is no clear explanation, they should test isolates from other patients as well for tolerance to rifampicin and/or performance in macrophage infection.

**Authors: We obtained preliminary results showing a better fitness upon incubation with RIF of the isolate of this patient (P1) after the first RIF exposure, suggesting the presence of RIF tolerant variants. Moreover, the fact that a non-fixed nsSNP (C 3280555 G) was revealed by RIF exposure of this isolate, was particularly interesting. However, we agree that the choice to focus on this particular patient was not clearly explained in the manuscript and, accordingly, we have added results of these RIF exposure experiments performed for all Mtb isolates obtained from delayed culture conversion patients to the supporting information section (Figure S1) and we revised the Results section (lines 145-156).**

5) The authors say that 4MBE has increased tolerance, but I do not think they are measuring tolerance. Tolerance is the ability to withstand the antibiotic even at high concentrations and it is generally measured using killing curves in which a high dose of the antibiotic is applied and the rate at which bacteria die is measured. Instead, the authors are measuring a mix of survival and growth ability even at sub-MIC concentrations. This kind of measurement is closer to relative fitness than to tolerance, in my opinion. I think that this shows that 4MBE has selective advantage but not tolerance, the authors should drop the term or show data that demonstrate said increase in tolerance. For their killing kinetics the authors say that they are taking measurements after 3, 5, 7 and 10 days of rifampicin exposure but they show only the results corresponding to day 7. Also, some of the rifampicin concentrations they are using are well above the MIC . This means that they could use those data to actually determine bacterial death rates and thus check for differences in tolerance. Why didn’t the authors do that?

**Authors: We agree that the methods used to explore RIF tolerance of the 4MBE variant were not the conventional ones. As requested, standard tolerance experiments were performed and a panel has been added to Fig 4. These experiments allowed to confirm that the 4MBE variant is more tolerant to RIF than the IMV. The Results section (lines 231-239) as well as the Material and Methods section (lines 536-540) have been revised accordingly.**

Part III – Minor Issues: Editorial and Data Presentation Modifications

6) The authors focus on intramacrophagic survival/replication of the 4MBE variant but give no data on overall replication or rationale about why that is not relevant.

**Authors: The overall replication of the variants in macrophage infection model was also explored as shown in the Figure below. However, as we observed the same profile, we decided to focus on intracellular bacteria which is more relevant for Mtb study. Yet these panels can be added to Figure 5 at Editor’s request.**

**Figure: Improved Mtb intra-macrophagic survival of the 4MBE variant.**

**Macrophages were infected by the IMV (black), the 4MBE variant (white) or a half-half mixture (50:50, grey) at a multiplicity of infection (MOI) of 10:1. (A) At 96 hours post-infection (hpi), macrophages were lysed after amikacin pre-treatment for intracellular CFU counting or all bacteria (in the supernatant and after macrophage lysis without amikacin pre-treatment) were recovered for total CFU counting. (B) Percentage of intracellular bacteria was deduced from CFU count. Values for each condition are the mean ± SD of five independent experiments. Means were compared using one-way ANOVA followed by Bonferroni correction.**

Reviewer #3:

Part I - Summary

Overall I think this is a worthwhile manuscript. It covers a topic of current interest, namely the role of unfixed genetic variants in the outcomes of tuberculosis disease. Strengths are the fact that it includes both clinical and genetic data, and then explores functional consequences of findings, which is often not done by equivalent papers.

1) A weakness of the clinical aspect is that no sequencing from control sequences are provided - it is therefore difficult to ascertain using the authors' sequencing/variant calling pipeline how common minor genetic variation is. Also, the paper would flow better if the authors were able to explain why the focussed on the mas minor variant, as currently the narrative jumps from the genetic section to the molecular biology section.

**Authors: We agree that control isolates from patients without delayed culture conversion are pertinent to support the conclusions made. Accordingly, a control cohort of 20 new patients with AFB+ pulmonary TB with proven culture conversion before 2 months of anti-TB treatment was added. Regarding initial isolates of the control cohort, 4/20 (20%) presented at least 1 unfixed mutation, among which 2 nsSNP, and 2 additional nsSNP from 2/20 (10%) isolates were revealed by RIF exposure. These results indicate a lower proportion of minor genetic variation in the control cohort compared to the delayed culture conversion cohort, supporting our conclusions. Table 2, summarizing patients and microbiological data, has been added in the revised manuscript. The Results section (lines 127-131 and 137-140), Discussion section (lines 350-352 and 362-364) and the Material and Methods section (lines 418-428) have been revised accordingly.**

**Concerning the focus on the mas minor variant, we obtained preliminary results showing a better fitness upon incubation with RIF for the isolate of this patient (P1) after the first RIF exposure, suggesting the presence of tolerant variants. Moreover, the fact that a non-fixed nsSNP (C 3280555 G, harbored by *mas* locus) was revealed by RIF exposure of this isolate was particularly interesting. However, we agree that the choice to focus on the *mas* variant is not clearly explained in the manuscript and, accordingly, we have added results of these RIF exposure experiments performed for all Mtb isolates obtained from delayed culture conversion patients to the supporting information section (Figure S1) and we revised the Results section (lines 145-156).**

Part II – Major Issues: Key Experiments Required for Acceptance

2) For the minor variant work, as a control of their variant calling pipeline, how many minor variants did they see in control sequences (e.g. baseline samples from patients who did culture-convert rapidly).

**Authors: As stated above,** **a control cohort of 20 new patients without delayed culture conversion was added, showing a lower proportion of minor genetic variation in the control cohort compared to the delayed culture conversion cohort, and the manuscript has been revised accordingly.**

Part III – Minor Issues: Editorial and Data Presentation Modifications

3) lines 38-40: I am not quite sure how these correspond to the samples listed in table 1. For the 9/12 patients with unfixed SNPs - which patients are these and which timepoint is referred to? Same for the 6/12 with nsSNPs.

**Authors: We agree that these points were not perfectly clear. Therefore, the abstract was clarified and updated with new delayed conversion patients (lines 40-42), as well as the corresponding Results section (lines 140-144) and the first paragraph of the Discussion section (lines 353-360) to clarify corresponding patients and isolates.**

4) lines 80: rephrase mandatory - strongly recommended, but some places still don't!

'as it is known to hamper treatment efficacy' - reword to make it clear 'it' is drug resistance, not drug resistance detection

**Authors: As requested, this sentence was rephrased in the revised version of the manuscript: “Anti-TB drug resistance detection is strongly recommended upon TB diagnosis, and mandatory in some high-income countries, as drug resistance is known to hamper treatment efficacy of first-line anti-TB drugs” (lines 83-86).**

5) Table 1: How often were cultures performed? Were cultures positive on a monthly basis until the time indicated? For those patients that died, after how many months' treatment did they die?

**Authors: RIF exposure experiments were performed once for each patient; the footnote of Table 1 has been clarified accordingly (line 158). Sputum samples were performed at diagnosis time, and 15 days, one months and two months after treatment initiation; we never observed culture conversion between time points in the follow-up of these 15 patients. Following this comment, the Results section (lines 121-122) and the Material and Methods section (line 417) have been clarified. Patient P6 died by secondary Aspergillus infection several months after treatment completion. Patient P8 died 2 months after the beginning of TB treatment. All these deaths were due to TB in a direct or indirect way, we therefore did not consider it relevant to add the above information to Table 1.**

6) Lines 135: was there a reason for focusing on this strain?

**Authors: We obtained preliminary results showing a better fitness upon incubation with RIF for the isolate of this patient (P1) after the first RIF exposure, suggesting the presence of tolerant variants. Moreover, the fact that a non-fixed nsSNP (C 3280555 G, harbored by *mas* locus) was revealed by RIF exposure of this isolate was particularly interesting. However, we agree that the choice to focus on the *mas* variant is not clearly explained in the manuscript and, accordingly, we have added results of these RIF exposure experiments performed for all Mtb isolates obtained from delayed culture conversion patients to the supporting information section (Figure S1) and we revised the Results section (lines 145-156).**

7) Line 302: as above, not sure what these numbers refer to

**Authors: As stated above, abstract, Results section and Discussion section have been clarified.**

8) Lines 430-438: how was bioinformatic analysis of targeted sequencing performed? What coverage depth was achieved and what variant calling thresholds were used?

**Authors: We agree that the Material and Methods sub-section “Bioinformatic analysis of Illumina data” need to be clarified regarding analysis of targeted NGS results. Regarding targeted NGS, a valid variant was called if the position was covered by a depth of at least 20 reads and supported by a minimum threshold rate of 0.1%. Depth of coverage of position of interest ranged from 10000 to 57000x. This Material and Methods section of the revised manuscript (lines 504-506) has been revised accordingly.**
